# Secondary Organic Aerosol from OH Oxidation of Acyclic Terpenes Is More Viscous and Less Volatile than That of Their Cyclic Analogs

**DOI:** 10.1021/acsestair.5c00226

**Published:** 2025-12-29

**Authors:** Sijia Liu, Claire E. Moffett, Gregory Vandergrift, Manish Shrivastava, Zezhen Cheng, Swarup China, Sergey A. Nizkorodov, Alla Zelenyuk, Celia L. Faiola

**Affiliations:** † Department of Chemistry, 8788 University of California, Irvine, Irvine, California 92697, United States; ‡ Atmospheric, Climate, & Earth Sciences Division, 6865 Pacific Northwest National Laboratory, Richland, Washington 99354, United States; § Environmental Molecular Sciences Laboratory, 6865 Pacific Northwest National Laboratory, Richland, Washington 99354, United States; ∥ Department of Ecology and Evolutionary Biology, University of California Irvine, Irvine, California 92697, United States

**Keywords:** secondary organic aerosol, SOA physical properties, sesquiterpenes, monoterpenes, viscosity, volatility, atmospheric chemistry

## Abstract

Biogenic volatile organic compounds (BVOCs), a dominant source of secondary organic aerosol (SOA) globally, exhibit emission rates and compositions that are plant species-specific and vary with environmental stressors. A common outcome of plant stress is increased emissions of acyclic terpenes. The paucity of information about acyclic terpene SOA chemistry contributes to uncertainties in predictions of SOA global loadings and impacts on Earth’s radiative budget, particularly in a changing climate where acyclic terpene emissions could become more prominent. This study compared properties of SOA derived from hydroxyl radical (OH) oxidation of acyclic and cyclic monoterpenes (β-ocimene, α-pinene) and sesquiterpenes (β-farnesene, β-caryophyllene). Single-particle mass spectrometry was used for assessing shape, density, and evaporation kinetics of size-selected SOA particles, and nanospray desorption electrospray ionization high-resolution mass spectrometry (nano-DESI-HRMS) was used to measure the molecular composition of SOA. Acyclic terpene SOA exhibited higher viscosity and lower volatility compared to cyclic terpene SOA, and had a greater volume fraction remaining (VFR) after ∼24 h of evaporation—approximately 1.3–1.6 times higher VFR than that of cyclic terpene SOA. Additionally, HRMS analysis revealed greater chemical diversity and higher fractions of extremely low-volatility compounds (56–62% ELVOC/LVOC) in acyclic terpene SOA compared to cyclic counterparts (25–37% ELVOC/LVOC). Our findings highlight the potential importance of accounting for acyclic terpene aerosol chemistry under conditions of plant stress to improve predictions of SOA loadings and impacts.

## Graphical abstract

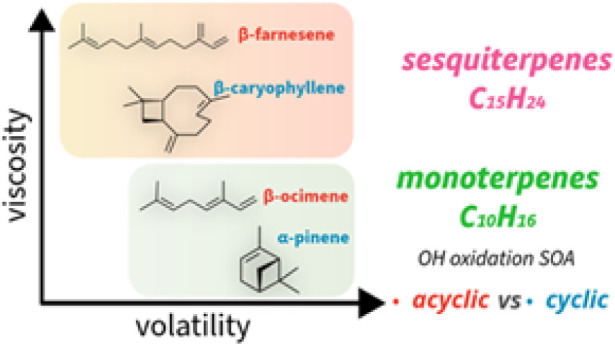

## Introduction

Secondary organic aerosol (SOA) forms in the atmosphere via oxidation of volatile organic compounds (VOCs), influencing cloud formation, earth’s radiative balance, and human health.^1–3^ SOA viscosity and volatility are important properties that alter particle evaporation and growth kinetics,^4,5^ which subsequently influence SOA mass loadings, atmospheric lifetimes, and the cloud condensation nuclei (CCN) budget. Additionally, these properties play important roles in ice nucleation,^6^ and atmospheric photochemistry,^7^ which are essential processes for predicting SOA loadings and radiative impacts. A substantial source of SOA arises from the oxidation of biogenic volatile organic compounds (BVOCs), such as isoprene, monoterpenes, and sesquiterpenes, which are secondary metabolites emitted from plants.^8–10^ These BVOCs play critical ecological functions, providing plant chemical defenses from insect herbivores and pathogens, and facilitating intraspecies and interspecies plant communication.^11–13^ The BVOC emission profiles of plants are species-specific and are modulated by environmental context, including plant stress.^14–16^ Plants are increasingly subjected to more frequent and severe stress conditions as climate rapidly changes,^17^ including, but not limited to, weather extremes, insect outbreaks, and drought, thus influencing plants’ BVOC emission rates and composition.^14,16,18–21^

One common plant stress response is elevated emissions of acyclic terpenes, especially acyclic sesquiterpenes, such as farnesene isomers.^22–25^ Despite their typical lower ambient concentrations compared to cyclic terpenes, the high reactivity of acyclic terpenes may enhance their role in atmospheric processes beyond what their abundance alone would suggest.^26^ For example, the rate coefficient for the reaction of β-ocimene with OH radicals is ∼ 4–5 times higher than that of α-pinene.^27–29^ In addition, Jardine et al. found that, despite the lower abundance of ocimene relative to α-pinene in the Amazon, its cumulative ozonolysis potential was approximately twice as high.^26^ There is also mounting evidence that acyclic terpene SOA chemistry is substantially different from that of their cyclic analogs, and depending on the oxidant, may lead to significant differences in physical properties of the resulting SOA. For example, oxidation of acyclic terpenes by ozone has been associated with reduced SOA formation potential compared to cyclic terpene analogs.^24,30–32^ The suppression of SOA mass yields during ozonolysis of acyclic terpenes was attributed to an increased level of fragmentation products compared to ozonolysis of bicyclic (e.g., pinenes) and monocyclic (e.g., limonene, phellandrenes) molecular structures.^30–32^ In seeming contrast to ozonolysis products, SOA generated by OH-initiated oxidation of VOC mixtures was found to have higher viscosity and increased propensity for liquid–liquid phase separation when the starting VOC mixtures contained more acyclic terpenes.^33–35^ Studies of acyclic terpene SOA chemistry are limited compared to their cyclic analogs and significant knowledge gaps remain, particularly with regard to systematic, comparative studies of the composition and physicochemical properties of SOA formed from cyclic versus acyclic terpene precursors.

The current study endeavors to bridge the knowledge gap in understanding the chemical composition, viscosity, and volatility of acyclic terpene SOA by comparing SOA properties from OH photooxidation of both cyclic and acyclic terpenes for two different compound classes – monoterpenes (C_10_H_16_) and sesquiterpenes (C_15_H_24_). The four terpenes used in this study were chosen for their prominent contribution to previously reported biogenic emission rates and to include representative compounds for both cyclic (α-pinene, β-caryophyllene) and acyclic (β-ocimene, β-farnesene) structures within the monoterpene and sesquiterpene classes.^26,30,31,36,37^ To compare the viscosity and volatility of SOA from cyclic/acyclic and monoterpene/sesquiterpene chemical systems, we utilized miniSPLAT, a single particle mass spectrometer capable of measuring the changes in particle size on the order of a monolayer with extremely high sensitivity and temporal resolution, facilitating the characterization evaporation kinetics and shape of SOA particles during evaporation.^38–40^ Nanospray desorption electrospray ionization high resolution mass spectrometry (nano-DESI HRMS) was utilized to examine the initial SOA molecular composition prior to evaporation. Through the integration of state-of-the-art measurements, this study offers new insights into the influence of precursor structure on SOA properties under OH-initiated oxidation. To the best of our knowledge, the current research is the first to systematically compare differences in composition and physical properties across cyclic and acyclic terpene SOA. These results can be used to refine the treatment of biogenic precursors in current regional and global models, particularly under stress-driven emission scenarios involving enhanced acyclic terpene release, and thereby inform continual model development with the aim of improving our understanding of how changing BVOC profiles influence atmospheric processes on a larger scale.

## Materials and Methods

### SOA Generation

SOA was generated in a cube-shaped 1 m^3^ fluorinated ethylene propylene (FEP) reaction chamber via OH photooxidation operated in batch-mode. To ensure the absence of residual particulate matter, the reaction chamber was flushed with nitrogen three times, followed by prefilling with zero air prior to each experiment. Subsequently, the VOC was introduced into the particle-free reaction chamber by injecting microliter quantities of α-pinene (Sigma-Aldrich, purity 98%), β-ocimene (Sigma-Aldrich, purity >90%, Lot # SHBL2427), β-caryophyllene (Sigma-Aldrich, purity >98%, Lot # BCBP1878 V), or β-farnesene (Supelco, purity >90%, Lot # BCCD9598) into a round-bottom flask. The flask was gradually heated to 75 °C while being flushed with 10 L/min of zero air to facilitate VOC evaporation into the chamber. Following the VOC injection, the OH radical precursor, 50 wt % hydrogen peroxide, was similarly injected into the 10 L/min air flow via the heated round-bottom flask and transported to the chamber. After introducing all the experiment components, additional clean air was added to the chamber until it reached its final volume of ∼ 1 m^3^. VOC photooxidation was initiated by turning on UV–B lights to start the photolysis of H_2_O_2_ and OH radical generation. All experiments were conducted under dry conditions, low nitrogen oxides (NO_*x*_) and at room temperature (24 ± 1 °C). The detailed experimental conditions and additional description are provided in the Supporting Information (SI, Table S1). Aerosol formation and growth were continuously monitored with a scanning mobility particle sizer (SMPS, TSI, Model 3082). After ∼ 2 h, the UV–B lights were turned off, particles were sampled *in situ* with miniSPLAT, and then all the residual SOA particles in the reaction chamber were collected on Teflon filters (Fluoropore, 0.2 μm PTFE membrane) at 20 L/min for subsequent offline composition analysis via nano-DESI HRMS, using methods developed and validated in previous studies^33–35,41^

### miniSPLAT Measurements

Following the formation of SOA, particle vacuum aerodynamic diameter distribution (*d*_*va*_), mass spectra, density, and shape were characterized using methods previously described in detail elsewhere.^38,40^ A differential mobility analyzer (DMA, TSI, model 3080) was employed to select monodisperse particles, typically with mobility diameters between 100 and 250 nm, and these particles were sampled by miniSPLAT (Figure S1).^4,42^ For each SOA chemical system, particle density and shape were evaluated across a range of particle sizes. For spherical particles, particle density (ρ_p_) was quantified from the relationship between measured *d*_*va*_ (from miniSPLAT) and mobility diameter (*d*_*m*_, from DMA), given by the equation $\rho_{p}=\frac{d_{va}}{d_{m}}\rho_{0}$ , where ρ_0_ stands for unit density (1 g/cm^3^). For aspherical particles the same equation yields particle effective density. Particle shape was characterized by examining the width of the *d*_*va*_ distribution, wherein a narrow symmetric distribution corresponds to spherical particles and a broader, often asymmetric, distribution indicates the presence of aspherical particles.^5,39^

SOA volatility was determined from particle evaporation kinetics under dry and humid conditions by transferring monodisperse particles (at # concentrations of ∼ 100–200 particles/cm^3^) from the SOA generation chamber through the inline denuder used to remove gas-phase organics into two stainless steel evaporation chambers at a flow rate of 0.3 L/min (Figure 1); one evaporation chamber was maintained at dry conditions and was partially filled with activated charcoal to continually strip the gas phase species, and the other evaporation chamber was maintained at 75% RH. Both evaporation chambers were kept at atmospheric pressure and room temperature. This was repeated for each of the acyclic and cyclic terpene SOA systems included in the study. The evaporation process was monitored in both wet and dry evaporation chambers over a ∼ 24 h window by periodically connecting the evaporation chambers to the miniSPLAT and measuring the particles’ *d*_*va*_ and mass spectra. During the initial stage of evaporation, samples were taken approximately every 30 min to 1 h, followed by one to two additional measurements overnight, per established methods.^4,42^ The evaporation kinetics data were fitted using a 7-bin volatility basis set (VBS), with the detailed fitting method described in the SI.^4,42,43^

**Figure 1:**
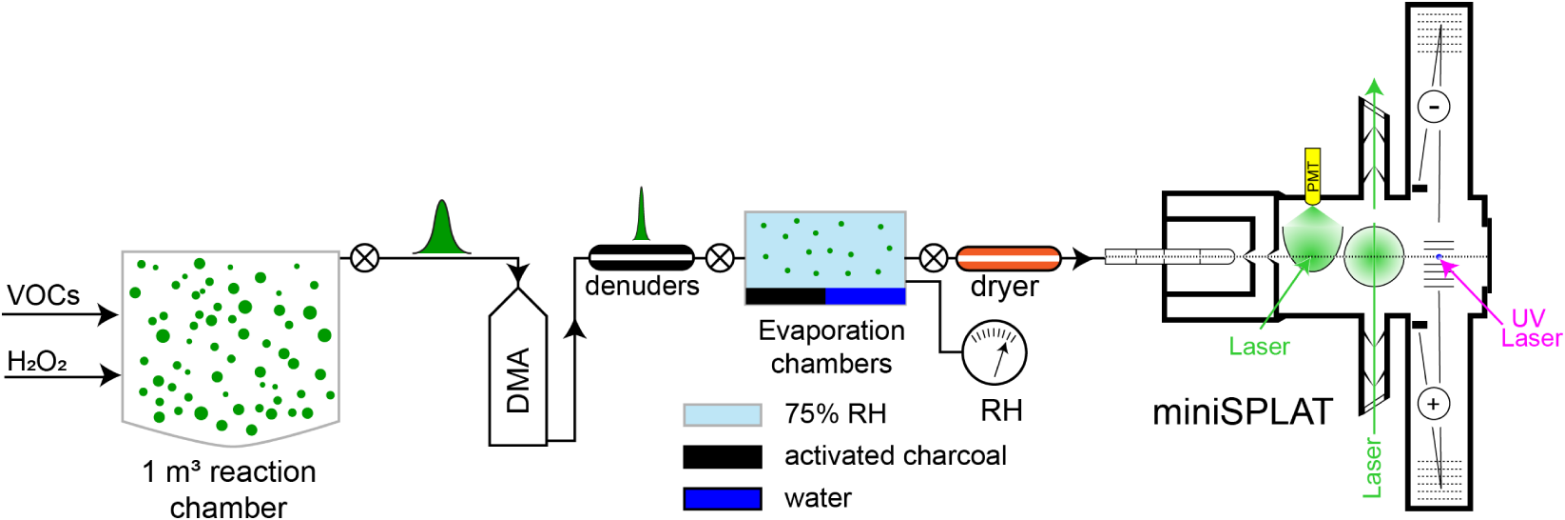
Schematic of the experimental setup for miniSPLAT measurements of evaporation kinetics. SOA from the reaction chamber were size-selected by a differential mobility analyzer (DMA), and then introduced into two evaporation chambers, where particle size distributions and evaporation kinetics were measured periodically by miniSPLAT over approximately 24 h.

### High-Resolution Mass Spectrometer Measurements

After the evaporation chambers were loaded with particles with desired mobility diameters, the remaining SOA in the reaction chamber was collected on a Teflon substrate (PTFE, 47 mm, Millipore) and immediately frozen at −20 °C. Samples were stored for no longer than 7 days under these conditions, minimizing chemical changes prior to analysis.^44^ The SOA filter samples were directly ionized from the filter *in situ* via a nano-DESI source, which was interfaced with a LTQ Velos Orbitrap high resolution mass spectrometer (Thermo Scientific, Waltham).^45,46^ The instrument was operated in negative ion mode over a *m*/*z* range of 100–1000. The data were extracted using FreeStyle 1.6 and the neutral molecular formula assigned by MFAssignR,^47^ as detailed in the SI.

### Volatility and Viscosity Prediction

The volatility and viscosity of SOA were predicted from the distribution of observed molecular formulas assigned from the HRMS data using established analytical procedures obtained from Li et al. and DeRieux et al.^48,49^ The detailed parameters and equations used are described in detail in the SI.

## Results and Discussion

### Particle Shape and Density

Physical properties of the particles formed in the reaction chamber were evaluated before the evaporation experiments were conducted. Particle shape and density can be characterized from miniSPLAT size-selected particle measurements by comparing the mobility and vacuum aerodynamic diameters as shown in Figure 2 where the different colored lines correspond to different sizes of particles selected with the DMA for the evaporation chamber and the *x*-axis indicates the vacuum aerodynamic diameter of the particles in the evaporation chamber measured with the miniSPLAT. Particle shape is qualitatively characterized based on the width and symmetry of the peak. where narrow distributions and symmetrical peak widths indicate more spherical particles. Particle density is calculated with the equation provided in the methods section, “miniSPLAT measurements.” This analysis revealed notable differences among the different SOA types (Figure 2). DMA-selected α-pinene SOA particles with a starting *d*_*m*_ of 165 nm displayed a *d*_*va*_ distribution peak at 205 nm, with a narrow line width and symmetrical peak shape indicative of a spherical shape (the blue line on Figure 2a). The 1.24 ± 0.01 g/cm^3^ density of α-pinene SOA derived from the measured *d*_*m*_ and *d*_*va*_ values is consistent with previous measurements, which range from 1.20 to 1.23 g/cm.^3,50,51^ Similar properties were observed for smaller (<250 nm *d*_*m*_) size-selected β-ocimene SOA particles, yielding particle density of 1.35 ± 0.01 g/cm^3^. Note, however, that the *d*_*va*_ size distributions of size-selected β-ocimene SOA particles with *d*_*m*_ of 250 nm exhibit a small but reproducible “tail” at smaller *d*_*va,*_ indicating the additional presence of a small fraction of aspherical particles, most likely formed by coagulation of smaller SOA particles, similar to previously published observations.^52^

**Figure 2:**
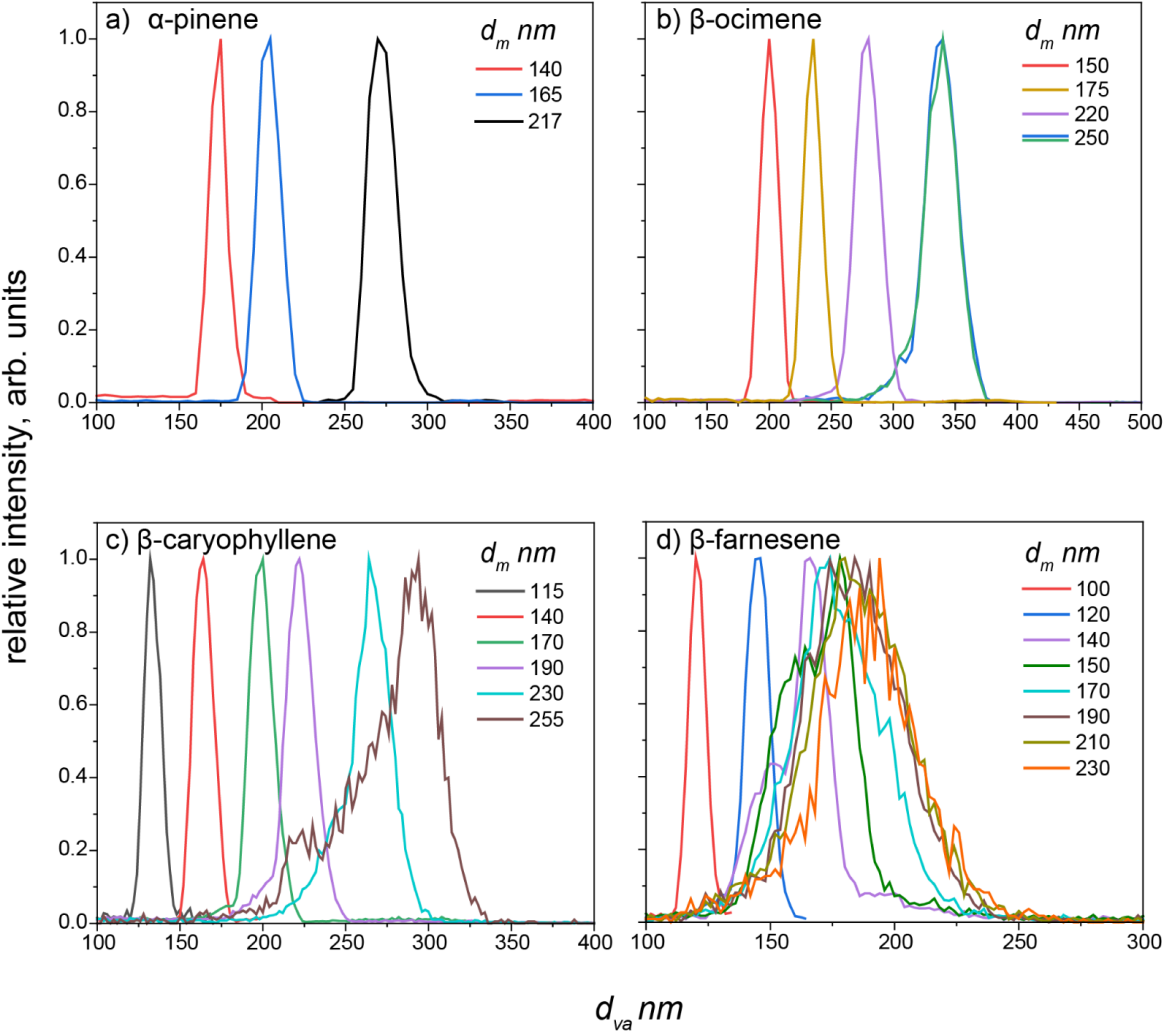
Vacuum aerodynamic diameter (*d*_*va*_) distributions from miniSPLAT for DMA size-selected particles of (a) α-pinene, (b) β-ocimene (250 nm was measured twice to verify the reproducibility of line-shape of *d*_*va*_ size distributions), (c) β-caryophyllene, and (d) β-farnesene OH oxidation SOA. The legend corresponding the mobility diameter (*d*_*m*_) for DMA-selected particles. The narrow distribution widths indicate that the particles are spherical in shape.

The *d*_*va*_ distributions for sesquiterpene SOA particles were narrow for smaller particles, but broader, and asymmetric for larger particles. Figure 2c shows the *d*_*va*_ distributions for size-selected β-caryophyllene SOA particles. Particles with smaller *d*_*m*_ (<∼200 nm) exhibit *d*_*va*_ distributions that are relatively narrow and symmetric, while particles with larger *d*_*m*_ have broader and more asymmetric distributions, indicating a shift from spherical to a mixture of spherical and aspherical particles with increasing *d*_*m*_. The presence of aspherical particles was observed for both sesquiterpene SOA, but it was more pronounced for β-farnesene compared to β-caryophyllene. In the case of β-farnesene SOA (Figure 2d), particles with *d*_*m*_ larger than 120 nm display broad distributions with a particularly broad distribution for particles with *d*_*m*_ greater than 190 nm, indicating a substantial fraction of aspherical particles. Larger particles form in the reaction chamber through the coagulation of smaller particles (Figure S2), and when particle viscosity increases, the coalescence relaxation time needed to the overcome surface tension after the collision and form spherical shapes becomes longer than the experimental time scale, leading to this size-dependent particle shape.^52–55^ Therefore, the evidence for increased aspherical particles in β-farnesene SOA suggests that their viscosity is higher than that of β-caryophyllene SOA. The higher abundance of aspherical particles at smaller sizes in β-farnesene SOA compared to β-caryophyllene SOA may be attributed to the acyclic structure and extra double bonds, which contribute to multifunctional oxidation products, and thus increase the overall particle viscosity of β-farnesene SOA. A detailed discussion of the SOA composition is provided in the offline analysis section.

The size-dependence of particle shape was additionally evaluated by looking at the trend in calculated particle densities/effective densities as a function of particle size. This approach supplements the qualitative assessment described in the previous paragraph. As the particle mobility diameter increased, there was a corresponding decrease in particle effective densities (Figure S3). Mathematically, this decrease means the vacuum aerodynamic diameter is becoming increasingly smaller than the mobility diameter at larger particle sizes, indicating that larger particles are more aspherical. The data were binned by spherical versus aspherical particles based on the broadness of the distribution and the density/effective density was calculated for each. The density of smaller spherical β-caryophyllene and β-farnesene SOA particles was calculated to be 1.17 ± 0.01 g/cm^3^ and 1.20 ± 0.01 g/cm^3^, respectively. However, the effective density of the larger, aspherical β-farnesene SOA particles with mobility diameter of 230 nm and *d*_*va*_ of 190 nm was 0.83 g/cm^3^, which is significantly lower than the density of spherical β-farnesene SOA particles.

### Particle Shape Evolution during Evaporation

For some of the SOA types (but not all), the shape of the particles in the evaporation chamber changed as the particles evaporated (Figure 3). This was evaluated by graphing the evolution of the vacuum aerodynamic diameter distributions (*x*-axis) over evaporation time (indicated with different colored lines). Recall, particle shape can be qualitatively characterized by the peak width and symmetry as described in the previous section. As the monoterpene SOA evaporated, the *d*_*va*_ distribution not only shifted to smaller values but also remained consistently narrow under both low and high RH (<10% and 75%, respectively) (Figure 3a and b show low RH conditions; Figure S3a and b show high RH conditions). This retention of the peak distribution over evaporation time indicates that the spherical shape of the particles was preserved throughout the evaporation process. The initial mobility size-selected particles that were used for the evaporation experiments were 165 nm for α-pinene SOA and 220 nm for β-ocimene SOA. These sizes were spherical when they were introduced to the evaporation chamber (see Figure 2 and associated text) and provided the required particle number concentrations (100–200 particles/cm^3^) needed to minimize complicating particle interactions while also enabling the instrument to measure particle evaporation kinetics for many hours. The shape size-dependence of the sesquiterpene SOA introduced additional challenges in conducting and interpreting the evaporation experiments.

**Figure 3:**
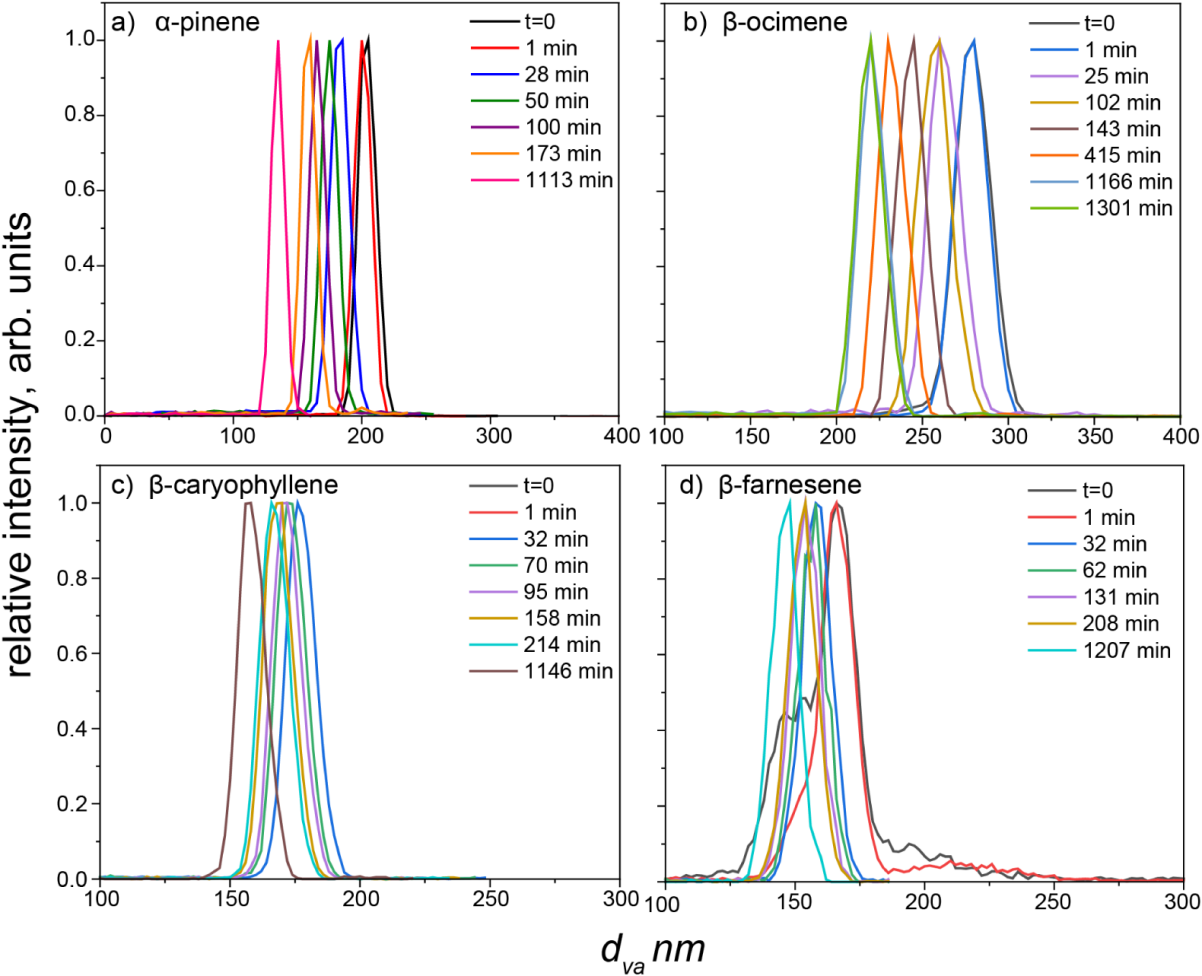
*d*_*va*_ distributions of mobility size-selected particles during evaporation under dry evaporation condition (RH < 10%) for: a) α-pinene SOA (*d*_*m*_ = 165 nm), b) β-ocimene SOA (*d*_*m*_ = 220 nm), c) β-caryophyllene SOA (*d*_*m*_ = 165 nm), and d) β-farnesene (*d*_*m*_ = 140 nm).

For the sesquiterpene SOA evaporation experiments, particle size selection targeted diameter ranges dominated by spherical particles to obtain narrow *d*_*va*_ distributions, thereby enabling the calculation of the volume fraction remaining (VFR). In the case of β-caryophyllene SOA, particles with *d*_*m*_ of 165 nm were spherical and had sufficient particle loadings to conduct the experiment. Throughout evaporation, β-caryophyllene SOA particles exhibited a consistently narrow *d*_*va*_ distribution under both low (RH < 10%) and high RH (75%) conditions indicating that the particle shape did not change over time (Figures 3c and S4c). In the case of β-farnesene SOA, particles with *d*_*m*_ of 140 nm represented a mixture of both aspherical and spherical shapes (Figure 3d) but also had high enough particle loadings to conduct the experiment. This mixture of particle shapes led to a broader, asymmetric *d*_*va*_ distribution; however, the peak maximum that corresponded to spherical particles remained clearly discernible in time-dependent *d*_*va*_ analysis (Figure 3d), allowing detailed study for the later evaporation kinetics. Notably, β-farnesene SOA particles underwent a rapid transition from aspherical to spherical shapes during evaporation in the dry evaporation chamber, coalescing within ∼ 30 min, as depicted in Figures 3d and S5. This shape transformation could be attributed to the thermodynamic drive toward minimizing surface energy, and the relaxation time scale can be used to estimate the SOA material viscosity – an analysis that could not be conducted with the other SOA types since they were spherical throughout evaporation. Frenkel et al. described the relationship between the coalescence relaxation time (*τ*) and viscosity (*η*), $\tau =\frac{d\eta }{\sigma }$ , where *d* represents the diameter of the particle (we used *d*_*m*_ in the calculation) and σ represents the surface tension.^56,57^ Using the observed relaxation time of 15 min and a surface tension σ=0.023–0.045 Ν m^–1^,^58^ we estimated the viscosity of β-farnesene SOA particles in the range of (1.4–2.8)×10^8^ Pa s, which corresponds to a semisolid state at room temperature.

### SOA Evaporation Kinetics

To further investigate differences in volatility and viscosity across SOA chemical systems, evaporation kinetics were measured under both dry and humid conditions. The evaporation kinetics show a two-stage process for all four SOA chemical systems (Figure 4), consistent with previous SOA evaporation kinetics measurements.^4,42^ Initially, a rapid decrease in SOA volume occurs within the first 2–3 h, followed by a more gradual reduction of volume over a 24-h duration. During the initial rapid evaporation phase, the particles lose predominantly more volatile compounds,^59^ leading to increased viscosity and decreased volatility of the residual particle, which contributes to slowing the evaporation rate.

**Figure 4:**
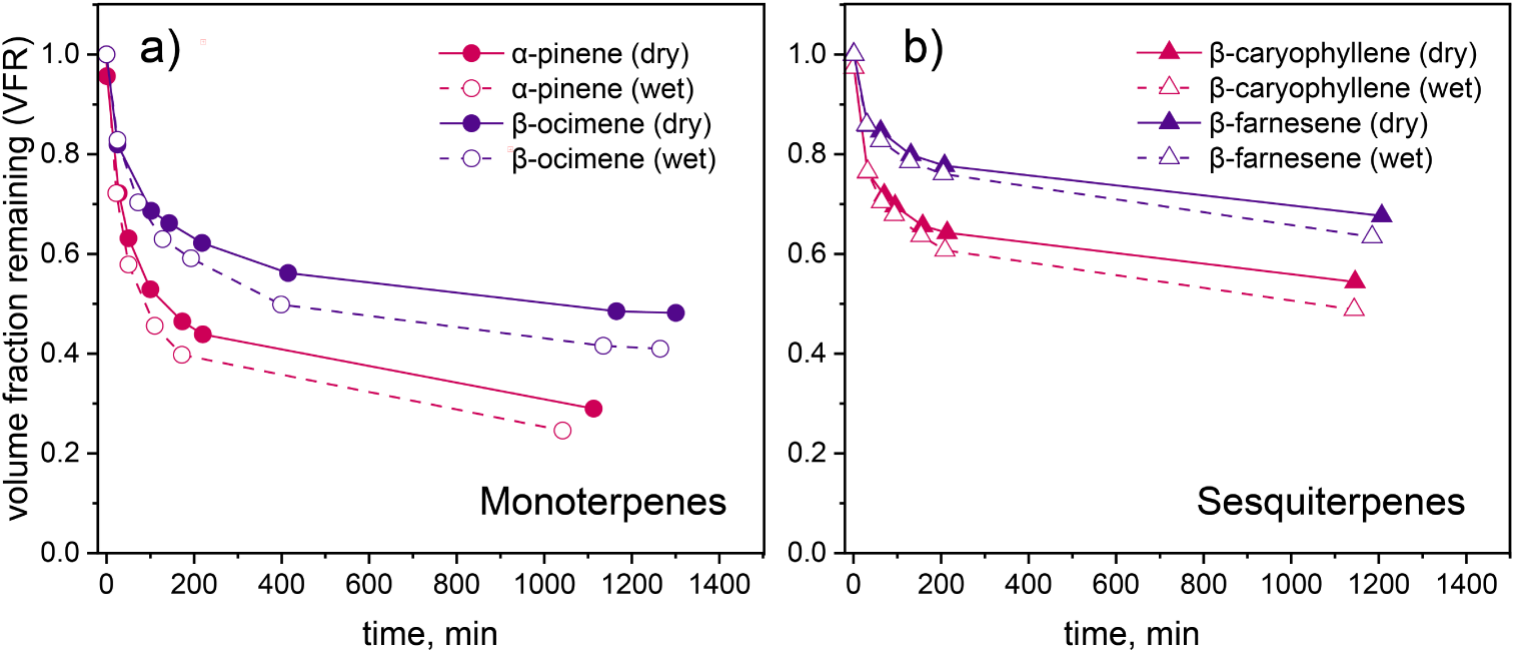
Evaporation kinetics of size-selected SOA particles generated from four different terpene structures. Panel a) shows evaporation behavior of α-pinene and β-ocimene SOA, while panel b) presents β-caryophyllene and β-farnesene SOA. Solid lines represent dry conditions (<10% RH), and dashed lines represent humid conditions (75% RH). Acyclic terpene traces are represented with purple, while cyclic terpene traces are represented with red.

We first discuss the particle evaporation kinetics under dry conditions (Figure 4). The evaporation kinetics of SOA particles from α-pinene, a cyclic monoterpene, exhibited a 54% decrease in volume within 200 min in the initial stage under dry conditions. This was followed by a slower rate of evaporation, ending with a 30% VFR at the final measurement (∼19 h), matching the previously reported VFR evolution for α-pinene under dry evaporation conditions.^42^ For β-ocimene, an acyclic monoterpene, the SOA particles demonstrated a 37% loss in volume during the initial 200 min, with 49% of the original volume persisting after a 21-h period. This VFR was higher than that of α-pinene SOA, implying a significantly lower volatility of β-ocimene SOA compared to α-pinene SOA.

Cyclic sesquiterpene SOA, represented by β-caryophyllene, displayed a 36% volume reduction initially (∼200 min), with a subsequent VFR of approximately 54% after ∼ 18 h. Notably, the VFR of β-caryophyllene SOA was only ∼ 5% more than β-ocimene SOA, despite the former precursor having a larger molecular weight, which would typically be expected to result in lower volatility and higher viscosity SOA.^60^ This highlights the importance of structural characteristics beyond molecular size alone for making predictions about SOA properties. The evaporation kinetics for β-farnesene SOA particles were characterized by an approximate 22% volume reduction within the first 200 min, concluding with a VFR of around 68% after 20 h, representing the least volatile SOA of all the chemical systems studied.

Under wet conditions (RH∼ 75%), we observed slightly higher evaporative losses compared to dry conditions across all SOA types (Figure 4). With increasing RH, particle viscosity decreases, and diffusivity within the bulk particle increases, facilitating greater evaporation for semivolatile organic compounds (SVOCs) that are trapped in highly viscous particles under dry conditions.^42,59^ It is noteworthy that regardless of the SOA type, the differences in evaporation kinetics between wet and dry conditions were quite small, with the final VFR differing by only ∼ 10%.

Evaporation kinetics were used to fit a 7-bin VBS as an additional metric for evaluating SOA volatility. This analysis revealed that, within the monoterpene and sesquiterpene families, SOA from acyclic terpenes displayed a greater fraction in the extremely low volatility organic compound (ELVOC) and low volatility organic compound (LVOC) ranges, compared to SOA from cyclic terpene, which had a higher fractional contribution in the SVOC range (Figure S6; Tables S4 and S5). Recall, acyclic terpene SOA also had the highest viscosity based on the shape measurements and coalescence times. Because of the known anticorrelation between viscosity and volatility of SOA,^61^ we expect the following order of viscosities for the SOA samples examined in this work: acyclic β-farnesene SOA > cyclic β-caryophyllene SOA > acyclic β-ocimene SOA > cyclic α-pinene SOA. This order is consistent with the observation of increased viscosity of SOA prepared from mixtures of VOCs which contain a higher proportion of sesquiterpenes.^33,34^ The observed evaporation kinetics also indicated a shift in the condensed organic mass into lower volatility ELVOC/LVOC bins, slower modeled evaporation rates, and extended particle lifetimes relative to current modeling parametrizations based mainly on cyclic terpenes. Exploring SOA formed under wet versus dry conditions and subsequently evaporated at different RH conditions^42,62^ is a natural follow-up for a future study but lies outside the scope of the present work.

### Chemical Composition of SOA from HRMS

Figure 5 shows nano-DESI HRMS mass spectra of all SOA types and Figure S7 shows the carbon atom distribution of the observed compounds. The predominant monomer identified in α-pinene SOA was C_10_H_16_O_5_, a formula commonly found in monoterpene SOA.^51,63,64^ The previously proposed chemical pathway for C_10_H_16_O_5_ includes RO_2_ termination with HO_2_, as discussed by McVay et al.^64^ The most intense dimer signal in α-pinene SOA was associated with C_20_H_34_O_*x*_ (x = 5–13), likely formed through the combination of two C_10_H_17_O_*x*_ RO_2_ species, which was thought to occur following OH addition and subsequent autoxidation.^65^

**Figure 5:**
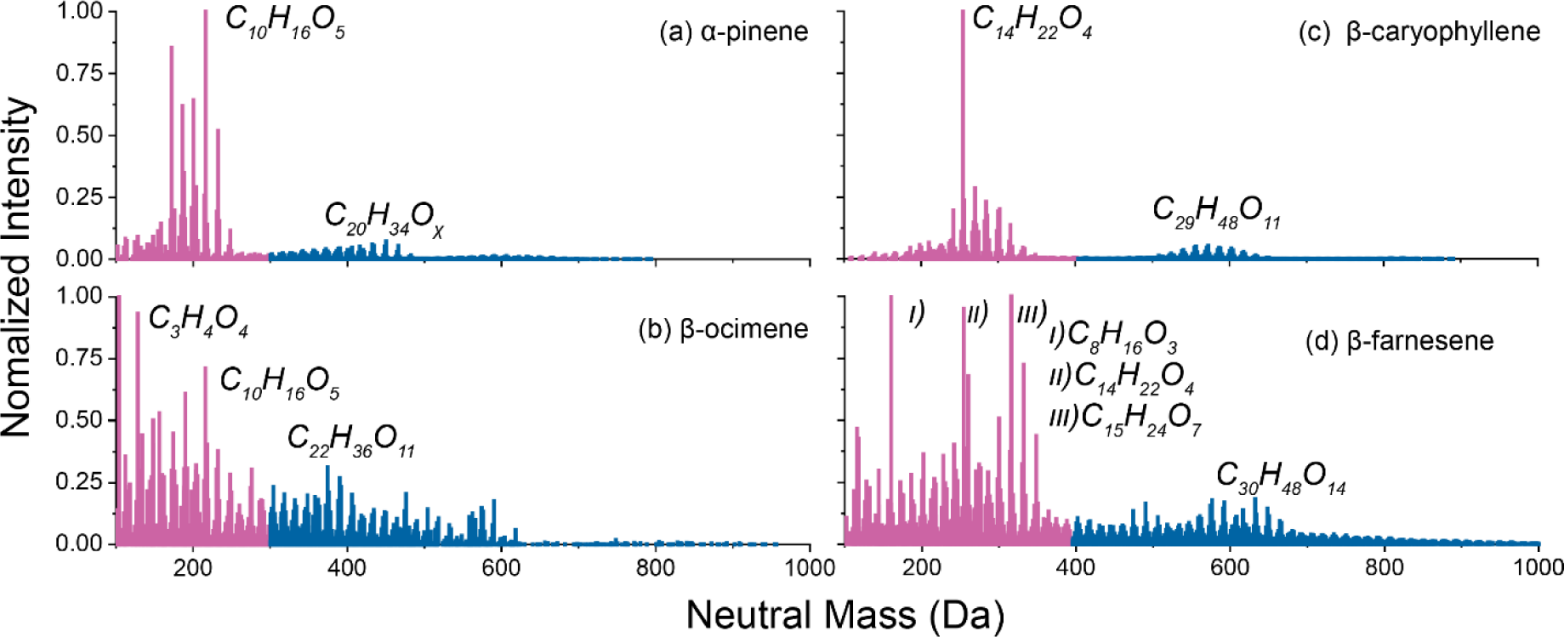
Nano-DESI mass spectra for SOA formed via OH photooxidation of a) α-pinene, b) β-ocimene, c) β-caryophyllene, and d) β-farnesene. Top monomer/dimer products are highlighted by red and blue based on their neutral mass. Note the difference of mass range for each SOA type.

For β-ocimene, we observed C_10_H_16_O_5_ as the most abundant monomer signal, which was the same for α-pinene SOA as they are isomeric compounds. The most intense peak detected for β-ocimene was attributed to the formula, C_3_H_4_O_4_, suggesting extensive fragmentation during oxidation. The most intense oligomer peak was C_22_H_36_O_11_, which was not found in α-pinene SOA. Gu et al. reported that β-ocimene oxidation in an oxidation flow reactor generates a substantial fraction of C_11_ products. Although C_11_ species were not the most intense peaks observed in the mass spectrum in our β-ocimene SOA, they were nevertheless detected (Figure S7b) and may serve as precursors to C_22_ species. These results indicate that, in contrast to α-pinene, β-ocimene follows distinct and more complex reaction pathways that require further investigation.

For β-caryophyllene SOA, the most pronounced peak was C_14_H_22_O_4_ which is likely β-caryophyllinic acid, consistently observed in both field and laboratory measurements, and a key atmospheric tracer for β-caryophyllene.^66–69^ The primary dimer identified in β-caryophyllene SOA was C_29_H_48_O_11_, presumed to be formed from the reaction of C_15_ and C_14_ RO_2_ species, which are two of the most abundant monomer families we observed.

The predominant peak detected in β-farnesene SOA was C_15_H_24_O_7_, a highly oxygenated molecule that has been attributed to sesquiterpene oxidation products in both field studies and laboratory experiments.^70,71^ Additionally, the most common dimer in β-farnesene SOA, C_30_H_48_O_14_, may originate from a combination of two C_15_H_24_O_*x*_ RO_2_ species, but the detailed formation pathway remains unexplored.

Overall, the mass spectra of SOA derived from cyclic terpenes and acyclic terpenes looked qualitiatively different based on distribution of the peaks (Figure 5). Mass spectra of cyclic terpene-derived SOA showed a distinct separation between monomer and dimer regions, whereas SOA from acyclic terpenes displayed a broader distribution across the mass spectrum, featuring higher peak signal for both dimeric and fragmented compounds than their cyclic terpene counterparts (Figure 5 and Figure S7). The Shannon diversity index was calculated for each SOA type to compare formula product diversity to evaluate this distribution with a more quantitative approach, where a larger index value indicates a higher level of chemodiversity.^72^ The acyclic terpene SOA exhibited higher diversity indices, with β-ocimene scoring 6.8 and β-farnesene scoring 6.7. In contrast, the cyclic terpene SOA had lower chemodiversity values, with α-pinene at 5.1 and β-caryophyllene at 5.7. These results are consistent with expected monomer reactivity, as acyclic terpenes contain more carbon–carbon double bonds than their cyclic analogs, providing more diverse reaction pathway and functional group addition. This therefore leads to increased chemical diversity of the oxidation products formed by both fragmentation and oligomerization reactions.^24,73^ To further assess differences in SOA composition, hierarchical clustering was performed on the various samples according to their annotated organic molecular composition (Figure S8). This analysis evaluated whether the cyclic/acyclic nature or the monoterpene/sesquiterpene classification of precursor is a better predictor of the resulting SOA molecular composition. The clustering revealed that samples with higher similarity in their mass spectra were grouped together. Notably, β-ocimene and β-farnesene clustered closely, indicating a similar formula composition among the acyclic terpene-derived SOA. This result underscores the dominant role of precursor cyclicity in determining the trends in SOA composition. These compositional patterns and clustering results also highlight the strong influence of precursor cyclicity on SOA chemical diversity. The distinct mass-spectral signatures associated with cyclic and acyclic terpene-derived SOA could be leveraged in future modeling and source-apportionment efforts to interpret and constrain field measurements.

Utilizing chemical formulas acquired from nano-DESI-HRMS analysis, we applied the methodologies outlined by Li et al. and DeRieux et al. to predict the volatility and viscosity of individual SOA compounds, as well as the integrated SOA composition.^48,49,61^ It is crucial to acknowledge, however, that this predictive approach utilizes the formula composition and does not incorporate considerations of chemical structure.^74^ This approach also does not account for different relative ionization efficiencies of the SOA species. We acknowledge that offline HRMS analysis and usage of single polarity may limit the quantitative resolution of certain compounds, such as labile peroxides. However, the main purpose of this estimation is to provide a qualitative explanation for the observed evaporation kinetics in miniSPLAT experiments, and an important point of comparison for previously published papers that have also used this estimation technique for other SOA systems.^34,35,68^

Figure 6 illustrates the distribution of SOA products across volatility categories. The analysis indicates that for SOA derived from cyclic terpenes (α-pinene and β-caryophyllene), approximately 25% and 37% of their signals, respectively, fell within regions corresponding to ELVOCs and LVOCs. In contrast, SOA mass spectra from their acyclic terpene counterparts, β-ocimene and β-farnesene, showed a higher prevalence of signals attributed to ELVOCs and LVOCs, at 62% and 56%, respectively (Figure 6). Among the ELVOC and LVOC detected, in addition to the oligomeric compounds that may be enhanced in smog chamber experiments due to relatively high VOC concentrations and thus greater RO_2_ availability, the SOA from acyclic terpenes also contained a larger fraction of ELVOC and LVOC present as monomeric/fragment (C ≤ 10 for monoterpene and C ≤ 15 for sesquiterpene SOA) components (0.8% for β-ocimene and 11% for β-farnesene) compared with SOA from cyclic terpenes (0.3% for α-pinene and 5% for β-caryophyllene). Because sesquiterpene precursors are less volatile than monoterpenes, their monomeric ELVOC/LVOC product fraction is correspondingly higher. The larger monomeric ELVOC/LVOC fraction in acyclic terpene SOA reflects their more efficient isomerization and autoxidation pathways compared to cyclic terpene, reducing volatility without changing the carbon skeleton, while fragmented ELVOC/LVOC indicate alkoxy radical pathways with C–C bond breakage. The average O:C ratios were quantified for different SOA types (Figure S9). The average O/C was similar for α-pinene (0.51), β-ocimene (0.53), and β-farnesene (0.51), but lower for β-caryophyllene (0.39).^75^ A plausible explanation is that β-caryophyllene’s complex double-ring structure may impose significant steric hindrance. This structure may reduce the molecule’s accessibility to autoxidation pathways, instead favoring RO_2_ pathways that produce less oxygenated and, therefore, more volatile products. The linear structure of acyclic terpenes results in less steric hindrance, which may facilitate pathways such as autoxidation, functionalization, and oligomerization which enhance the LVOC and ELVOC formation.

**Figure 6:**
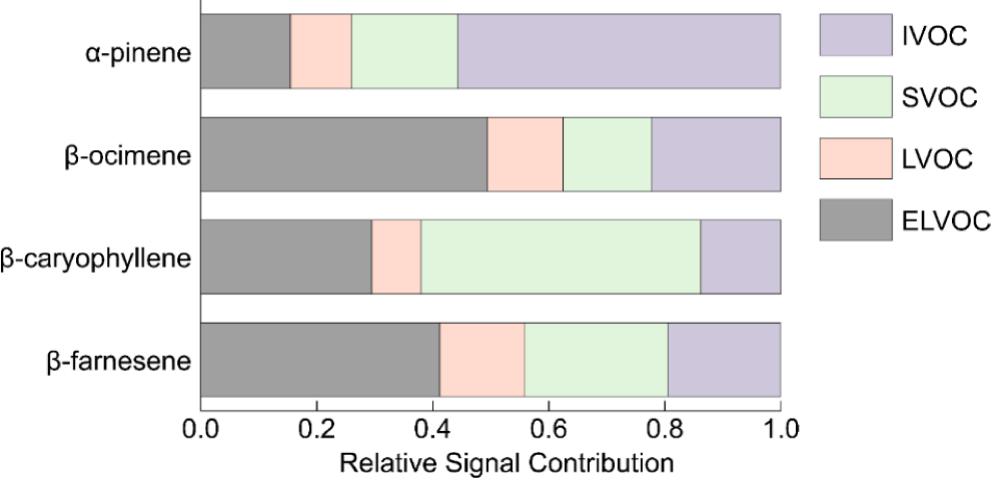
Relative contributions of each volatility category (ELVOCs (C_0_ < 3 × 10^–4^ μg m^–3^), LVOCs (3 × 10^–4^ < C_0_ < 0.3 μg m^–3^), SVOCs (0.3 < C_0_ < 300 μg m^–3^), and IVOCs (300 < C_0_ < 3 × 10^6^ μg m^–3^)) to the overall composition of SOA from OH oxidation of α-pinene, β-ocimene, β-caryophyllene, and β-farnesene.

Using the observed chemical formulas, we predicted the viscosity of SOA from different types of VOCs under varying RH levels, as illustrated in Figure 7. At 0% RH, all SOA systems were predicted to be highly viscous.^76^ With an increase in RH, the viscosity of all SOA systems decreased. However, the magnitude of this reduction varied depending on the chemical system. Specifically, within the monoterpene and sesquiterpene families, the calculation suggested that acyclic terpene SOA would remain viscous and may have a longer lifetime under a broader range of RH conditions in the lower atmosphere, from a compositional perspective. We emphasize that this analysis is not intended to predict SOA viscosity in a quantitative way, as absolute viscosity also depends on detailed molecular structure and interactions, but the relative ordering of SOA viscosity aligned well with our observations from evaporation kinetics experiments. Overall, the volatility and viscosity trends predicted from the mass spectra qualitatively matched the observations from evaporation kinetics: acyclic terpene SOA exhibited higher viscosity and lower volatility compared to SOA from the cyclic terpene analogs.

**Figure 7:**
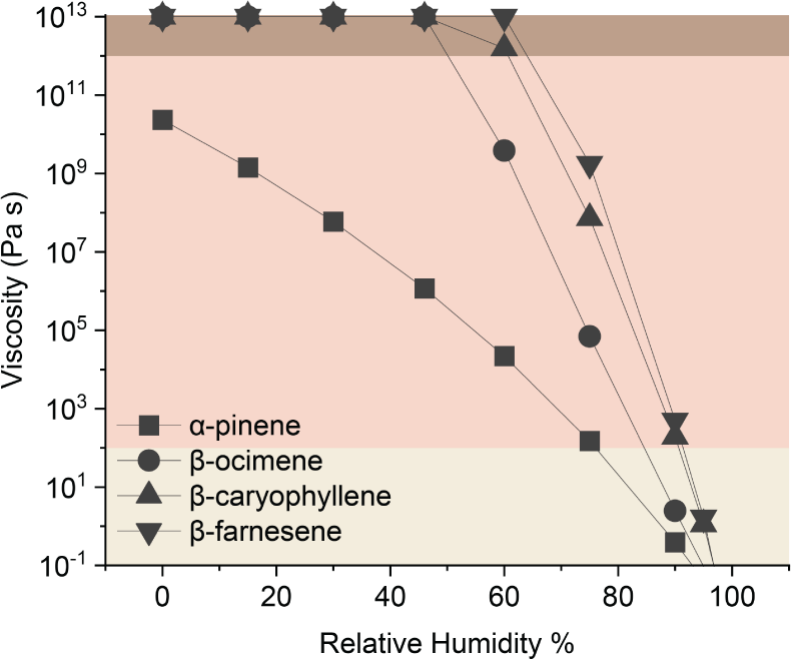
Predicted particle-phase viscosity of SOA derived from four different VOCs (α-pinene, β-ocimene, β-caryophyllene, and β-farnesene) as a function of water content. Viscosity predictions are based on the molecular formulas of assigned compounds. Shaded regions indicate viscosity benchmarks. From the darkest color, the viscosity ranges from solid, semisolid, to liquid.

## Conclusions

By systematically comparing the particle shape and evaporation kinetics of SOA derived from OH oxidation of different terpenes, we have elucidated a clear trend in SOA viscosity as follows: α-pinene < β-ocimene < β-caryophyllene < β-farnesene, and a corresponding inverse trend in volatility: α-pinene > β-ocimene > β-caryophyllene > β-farnesene. In fact, the volatility and viscosity of β-ocimene SOA are comparable to those of β-caryophyllene SOA, suggesting that acyclic terpene can produce low-volatility products beyond the expectation based solely on their molecular weight. Further analysis of formula composition has shown SOA from OH oxidation of acyclic terpenes shows greater chemical diversity than that from cyclic terpenes, reflecting more complex oxidation pathways enabled by additional double bonds. For instance, OH can add at two different positions in α-pinene but at six in β-ocimene. The number of ways in which the resulting RO_2_ can isomerize and RO radicals can fragment is also larger for acyclic compounds, leading to a greater diversity of products. The large portion of oligomers of acyclic terpene SOA contributes to their elevated viscosity and diminished volatility in comparison to cyclic terpene SOA.

These results have important implications. First, the physical properties of SOA from acyclic terpenes suggest that these compounds may influence aerosol lifetime, transport, and phase behavior in ways not fully captured by current models that focus on cyclic monoterpenes like α-pinene. For example, increased viscosity can slow down diffusion within particles, reduce rates of heterogeneous reactions, and inhibit mixing. Lower volatility can shift partitioning toward the particle phase and modify particle loading and their atmospheric lifetime. Additionally, as plants respond to environmental stressors, such as drought, heatwaves, and herbivore attacks, their emission profile is expected to change. This evolution in BVOC profiles could ultimately shift the physicochemical properties of atmospheric SOA. Together, we highlight a need to revisit how SOA precursors are represented in regional and global models. Without including more structurally diverse BVOC, current models may overlook pathways and feedback associated with other terpene structures, including those that may become more prominent in a changing environment which are necessary for estimation of SOA formation and mass loading. Incorporating a broader range of BVOC structures, especially under stress-driven emission scenarios, will improve predictions of aerosol formation and transformation, aerosol-cloud interactions, and the long-term atmospheric impacts of SOA. We suggest further study of acyclic terpenes through different SOA formation pathways with other oxidants, such as ozonolysis pathway, as well as their physicochemical properties and aerosol yield.

## Supplementary Material



## Data Availability

The data sets used to generate the figures and tables in this work are available in the Dryad Digital Repository at 10.5061/dryad.931zcrk0c.

## Notes

Tabular summary of experimental conditions, description of nano-DESI-HRMS and mass spectrometric data analysis, description of VBS fitting of evaporation data, description of volatility and viscosity estimation from the observed formula distribution, and additional figures and tables illustrating SOA particle properties, chemical composition, and volatility (PDF)
