# Supplementary material for: Secondary Organic Aerosol from OH Oxidation of Acyclic Terpenes Is More Viscous and Less Volatile than That of Their Cyclic Analogs
Source: ACS EST Air. 2025 Dec 29;3(1):83–94. doi: 10.1021/acsestair.5c00226 (PMC12797235; doi:10.1021/acsestair.5c00226)
Supplement: Supplementary file 1 [file ea5c00226_si_001.pdf]

Supporting information for

Secondary Organic Aerosol from OH Oxidation of Acyclic Terpenes is More Viscous and Less Volatile Than That of Their Cyclic Analogs

Sijia Liu,[1,3] Claire E. Moffett,[2] Gregory Vandergrift,[3] Manish Shrivastava, Zezhen Cheng,[3]Swarup China,[3] Sergey A. Nizkorodov,[1] Alla Zelenyuk,[2] Celia L. Faiola[1,4]

1.Department of Chemistry, University of California, Irvine, Irvine, California 92697, United State

2.Atmospheric, Climate, & Earth Sciences Division, Pacific Northwest National Laboratory, Richland, Washington, 99354, United States

3.Environmental Molecular Sciences Laboratory, Pacific Northwest National Laboratory, Richland, Washington 99354, United States

4.Department of Ecology and Evolutionary Biology, University of California Irvine, Irvine, California 92697, United States

Table S1. The experimental conditions and particle size selected for evaporation kinetics.

Experiment date	VOC type	Size selected for evaporation kinetics (d_m, nm)	Calculated density (g/cm^3)	Targeted VOC concentration (ppb)	GC-estimated VOC concentration (ppb)
08/23/22	α-pinene	165	1.24 ± 0.01	285	N/A
08/25/22	β-ocimene	220	1.35 ± 0.01	283	N/A
09/01/22	β-ocimene	150		50	N/A
09/06/23	β-caryophyllene	165	1.17 ± 0.01	53.4	N/A
09/07/23	β-caryophyllene	130	1.16 ± 0.01	32.0	15.0
09/08/23	β-farnesene	140	1.20 ± 0.01	48.2	N/A
09/11/23	β-farnesene	135	1.19 ± 0.01	40	11.7

Nano-DESI-HRMS sample and MFAssignR analysis.

Due to the limited collected SOA mass, we utilized nano-DESI-HRMS for offline compositional analysis. A general description of the nano-DESI instrument can be found in Roach *et al.*, 2010.[1] The nano-DESI interface capillary (Polymicro Technology L.L.C., Phoenix, USA) was coupled to the inlet of the mass spectrometer, with two capillaries aligned at a 90° angle. The capillary features an outer diameter of 150 µm and an inner diameter of 50 µm. The secondary capillary, approximately 1.5 cm in length, was

positioned roughly 1 mm away from the MS inlet. A solvent mixture of 7:3 acetonitrile: water (Optima LC-MS grade, Fisher Chemical, USA) was delivered at a flow rate of 500 nL/min through the primary capillary by a syringe pump. This arrangement facilitated the formation of a liquid interface between the capillaries on the sample surface, allowing for the continuous transfer of extracted analytes to the orbitrap spectrometer. A high voltage of -3.5 kV was applied to the syringe needle in negative ion mode. The capillary temperature was maintained at 275°C. The maximum ion injection time and the Automatic Gain Control (AGC) target were set to 500 ms and 5×10^5, respectively. The mass spectrometer was configured to acquire mass spectra from *m/z* 100-1300 at a mass resolution of 100,000 (at *m/z* 400). Via a XYZ Zaber sample stage and custom LabVIEW software, the sample was scanned along the XY plane under the nano-DESI liquid junction at 75 μm/s, ensuring consistent and fresh sample desorption for consecutively collected HRMS scans (100 scans).

MFAssignR was employed for assigning formulas to detected *m/z* values;[2] comprehensive details about the software are provided in previous publications.[3,4] Noise and isotope features were filtered out from the mass list, with the final assignments to formulae $C_xH_yO_z$ being made with an accuracy of 4 ppm. The processing of both blank and sample data followed a uniform protocol. Subsequently, signals present in both the blank and sample were removed if the signal in the sample was not at least 10 times greater than that in the blank. We analyzed both Teflon filter blanks and solvent blanks using the same nano-DESI protocol as for the samples. Peaks present in the blanks were removed unless the corresponding signal intensity in the sample was at least an order of magnitude (≥10×) higher than in the blank. Only these blank-screened peaks were retained for subsequent data analysis and interpretation.

Chemodiversity was estimated using Shannon's index (H):[5]

$$H = -\sum_{i=1}^{S} p_i \times \ln p_i$$

and in this formula, p_i represents the normalized intensity of individual formula product, and S is the species richness.

7-bin Evaporation Kinetics Fits and Modeling.

The methods used to fit and model evaporation kinetics have been described in detail elsewhere and are summarized here.[6–8] Measured evaporation data were fit with a bi-exponential decay function of the form $VFR=Ae^{-at} + Be^{-bt}$, where t is the evaporation time. In brief, time-dependent evaporation of multicomponent single particles was simulated using a seven-bin VBS, assuming that activated charcoal removes gas-phase

organics as they evaporate. Particles were treated as uniform and non-interacting. The initial kinetic equations simulate single-particle evaporation given an assumed VBS, and the VBS mass fractions are iteratively adjusted until the modeled kinetics match the measurements. Volatility bins span effective saturation concentrations from $1x10^{-4}$ to $1x10^{2}$. The lower boundary for the lowest-volatility bin was defined at this value because the compounds with this and lower effective saturation concentrations remain in the particle phase at room temperature within the timescale of the experiments (~24 hours). This means we are unable to resolve compounds in different bins at volatilities lower than this even though other papers have defined ELVOC as $c^* < 3x10^{-5}$.

Viscosity and Volatility Estimation.

Glass transition temperature (T_g) is the point at which the state of SOA changes from viscous semisolid to a glassy solid state, and T_g of SOA can be predicted using the molecular formulae from the mass spectrometry:[9]

$$T_g = \left(n_C^0 + \ln(n_C)\right)b_C + \ln(n_H)b_H + \ln(n_C)\ln(n_H)b_{CH} + \ln(n_O)b_O + \ln(n_C)\ln(n_O)b_{CO},$$

where n_C^0 stands for reference carbon number, while $b_C,\ b_H,\ b_{CH},\ b_O$, and b_{CO} represent the contribution of carbon, hydrogen, carbon-hydrogen interaction, oxygen, and carbo-oxygen interaction to T_g, and the values are listed in Table S2.

Chemical compound classes and parameters for estimating glass transition temperature, as described by DeRieux et al.[9]

Classes	n_C^0	b_C	b_H	b_{CH}	b_O	b_{CO}
CH	1.96	61.99	-113.33	28.74		
CHO	12.13	10.95	-41.82	21.61	118.96	-24.38

Under dry conditions, the T_g values for a mixture of SOA were derived using a linear approach through the Gordon-Taylor method, which assumes Gordon-Taylor constant (k_{GT}) equal to 1, as expressed by the formula below.[10,11]

$$T_g = \sum_i w_i T_g$$

Here, w_i indicates the mass fraction of the ith component, derived from mass spectra peak abundances. In humid conditions, the presence of water alter the T_g, which can be calculated for a water-organic mix using Gordon–Taylor equation:[12]

$$T_{g\,(w_{org})} = \frac{\left(1 - w_{org}\right) T_{g,w} + \frac{1}{k_{GT}} w_{org}\, T_{g,org}}{\left(1 - w_{org}\right) + \frac{1}{k_{GT}} w_{org}}$$

where w_{org} is the mass fraction of organic components, $T_{g,w}$ is the glass transition temperature of water (136 K), and k_{GT} is the Gordon-Taylor constant, set here to 2.5.[12,13]

The effective hygroscopicity parameter (κ) was assumed to be 0.10 for monoterpene-derived SOA and 0.05 for sesquiterpene-derived SOA due to the previously observed hygroscopicity differences and estimated values,[14–19] consistent with values reported in previous studies. These values were used in the computation of the mass concentrations of SOA (m_{SOA}) and water (m_{H2O}):

$$m_{H_2O} = \frac{\kappa \rho_w m_{SOA}}{\rho_{SOA}(\frac{1}{a_w} - 1)}$$

Here, ρw and ρSOA denote the densities of water and SOA, measured at 1 g cm^{-3}and 1.4 g cm^{-3} respectively, whereas aw symbolizes water activity, which is computed as a_w=100/RH.

Furthermore, the dependence of viscosity on temperature was determined through the Vogel-Tammann-Fulcher (VTF) equation:

$$\log(\eta) = -5 + 0.434\, \frac{T_0 D_f}{T - T_0}$$

In this equation, T_0 stands for the Vogel temperature, which can be calculated using $T_0 = \frac{39.17\, T_g}{D_f + 39.17}$, D_f is the fragility parameter, quantifies the deviation from Arrhenius behavior and was postulated to be 10.[9]

To calculate the volatility distribution of SOA, the parameterization approach by Li et al. was employed.[20] This method allows for the estimation of the saturation mass concentrations (C_0) of pure compounds using the formula:

$$log_{10} C_0 = \left(n_C^0 - n_c\right) b_c - n_O b_O - 2\, \frac{n_C n_O}{n_C + n_O}\, b_{CO}$$

Here, n_{C0} represents the reference carbon number, while n_C and n_O indicate the counts of carbon and oxygen atoms, respectively. The coefficients b_C, b_C, and b_{CO} are provided in Table S3. Although this formulaic approach is suited for estimating volatility in

isomeric mixtures, it's important to note that the absence of detailed structural information introduces a degree of uncertainty to these estimations.

Table S3. Classes of chemical compounds and saturation mass concentration parameterization described by Li et al..[20]

Classes	n_C^0	b_C	b_O	b_{CO}
CH	23.8	0.48861		
CHO	22.66	0.4481	1.656	-0.7790

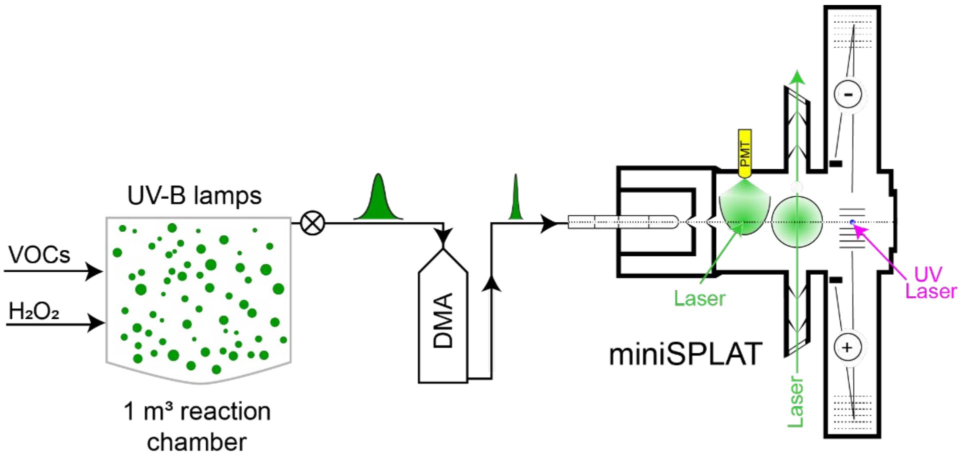

Figure S1. Schematic of the experimental setup for miniSPLAT measurements of density and shape of size-selected particles generated in the reaction chamber by photooxidation.

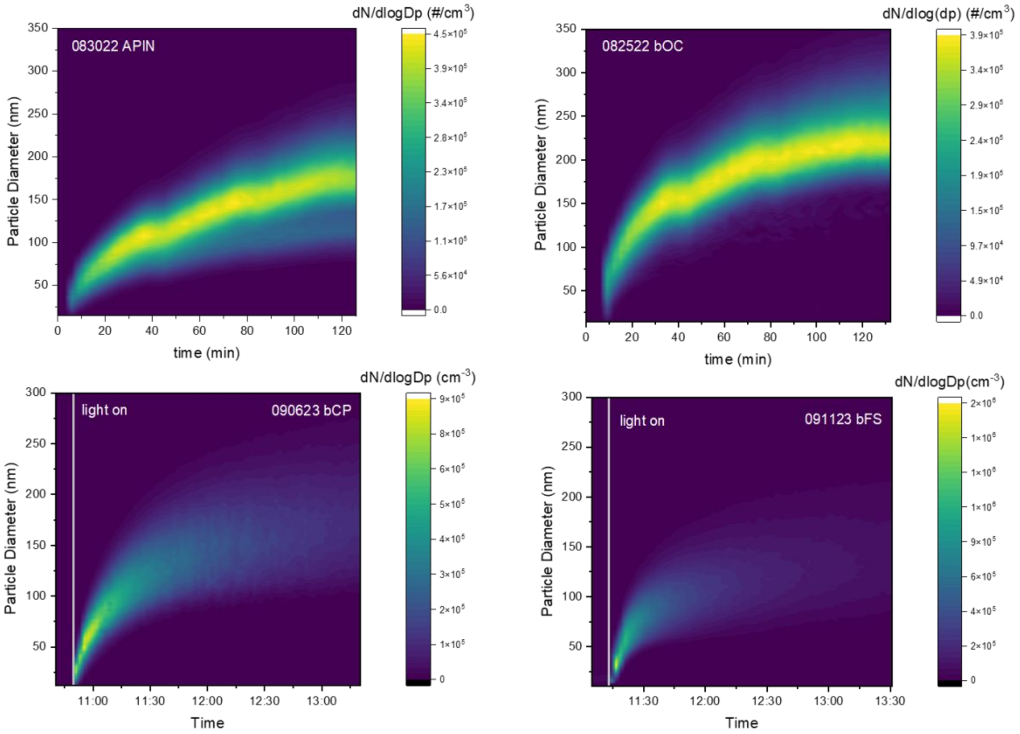

Figure S2 The observed evolution of particle mobility diameter during SOA growth, with color representing the dN/dlogDp. The small gaps observed for monoterpene SOA resulted from VOC cartridges sampling, with the UV-lights being temporarily off.

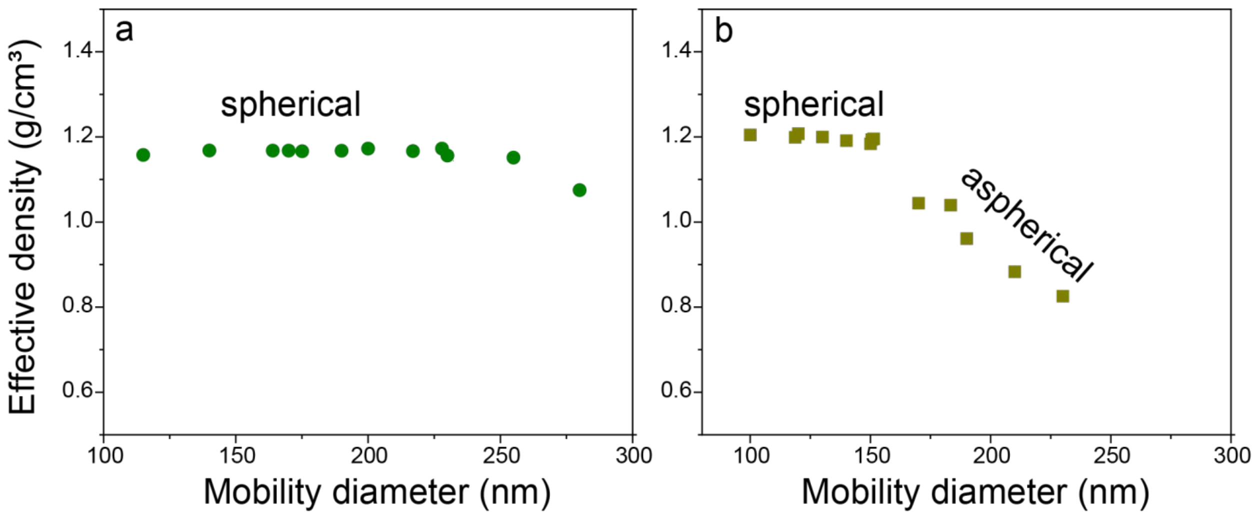

Figure S3 The relationship between density/effective density and mobility diameter (d_m) for (a) β-caryophyllene, and (b) β-farnesene SOA size-selected particles, indicating the small particles are spherical, while larger particles become aspherical and have lower effective densities.

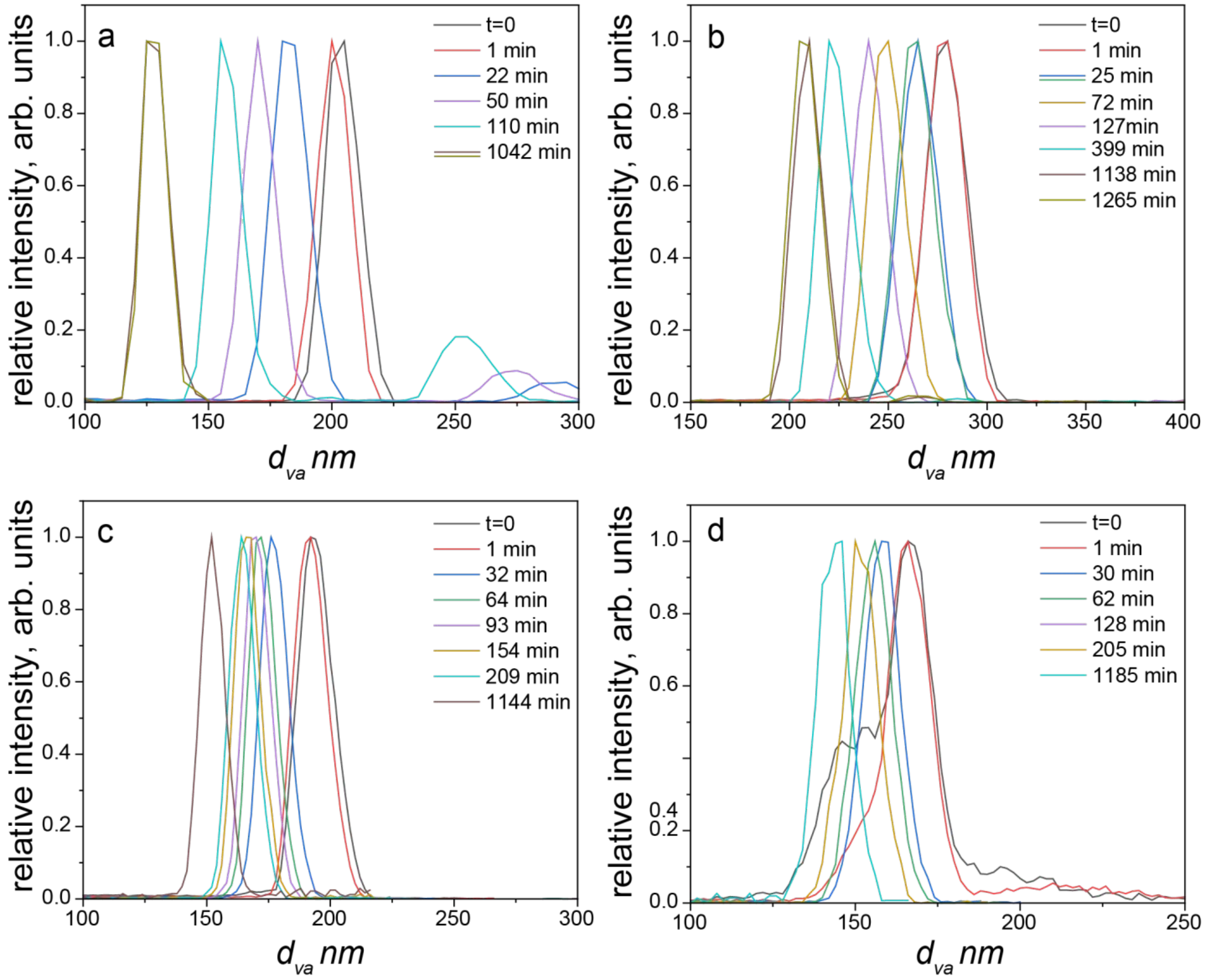

Figure S4 The d_{va} distribution as a function of evaporation time of (a) α-pinene (doublet particles observed), (b) β-ocimene, (c) β-caryophyllene, and (d) β-farnesene SOA during wet evaporation.

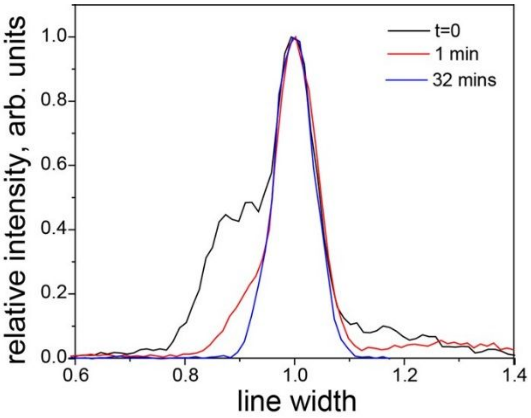

Figure S5 Linewidths of d_{va} distributions for β-farnesene SOA particles measured at 0-, 1-, and 32-minutes during evaporation, illustrating changes in particle shape and distribution broadness over time.

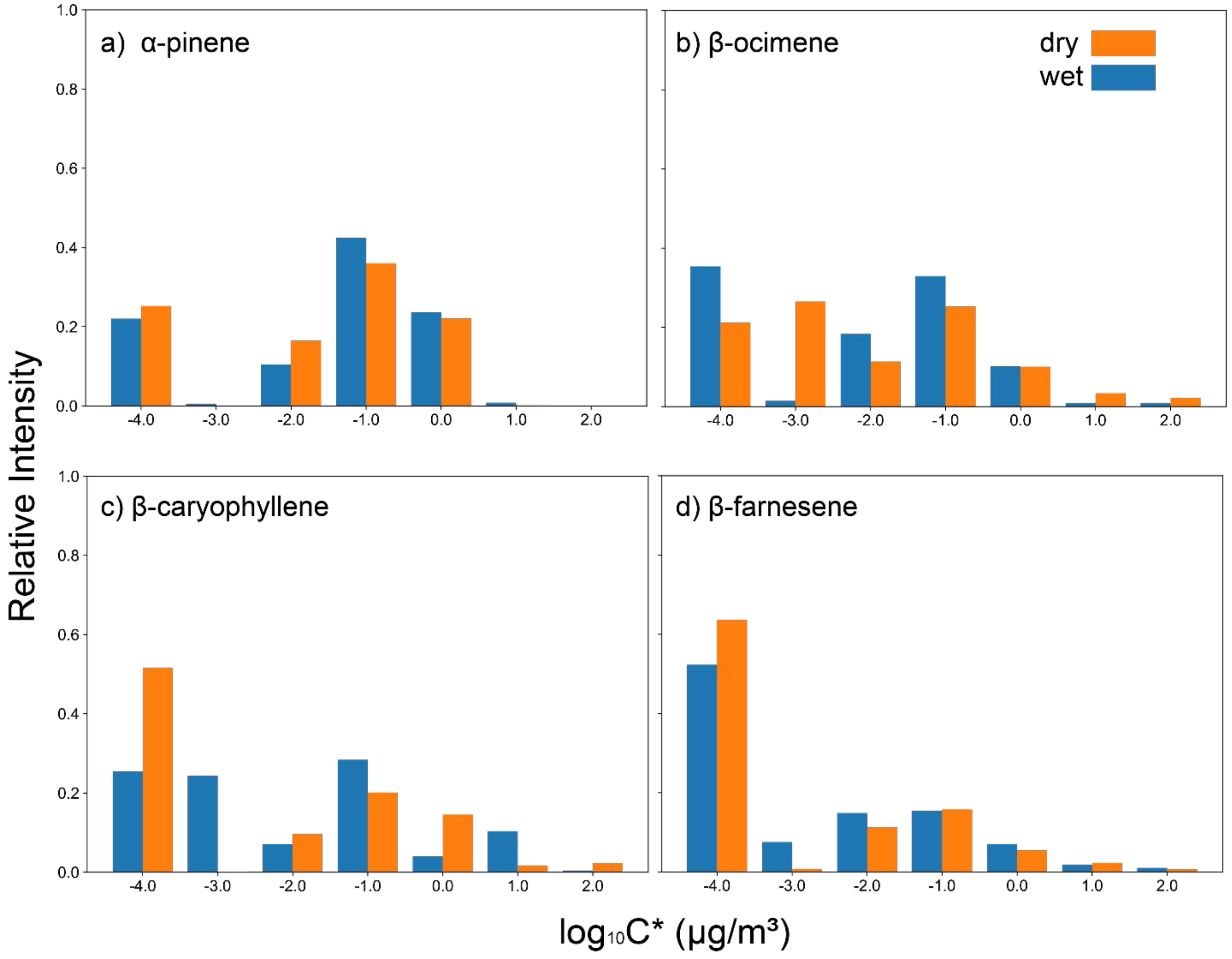

Figure S6 Comparison of wet and dry fraction value across 7 volatility bins on a logarithmic scale, derived from evaporation kinetics.

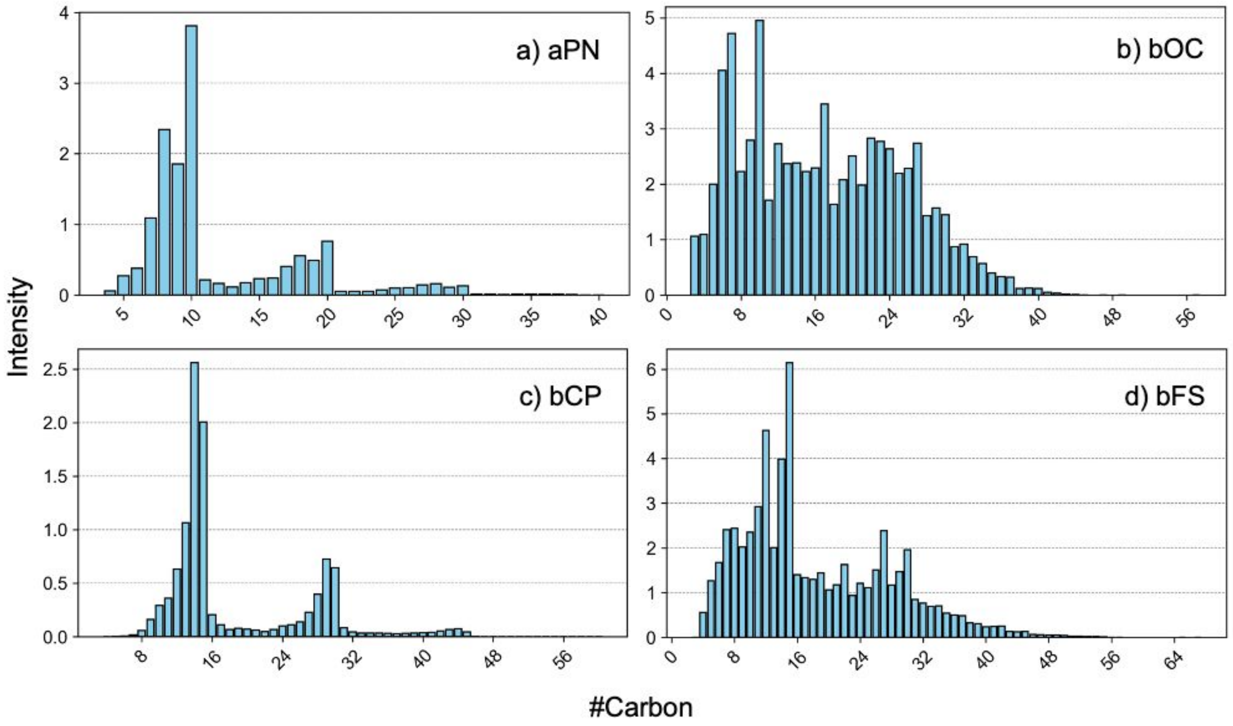

Figure S7 Total intensity distribution by carbon number for (a) α-pinene(aPN), (b) β-ocimene(bOC), (c) β-caryophyllene (Bcp), and (d) β-farnesene SOA (bFS). This bar chart represents the summed intensities for each carbon number, derived from nano-DESI-HRMS measurements.

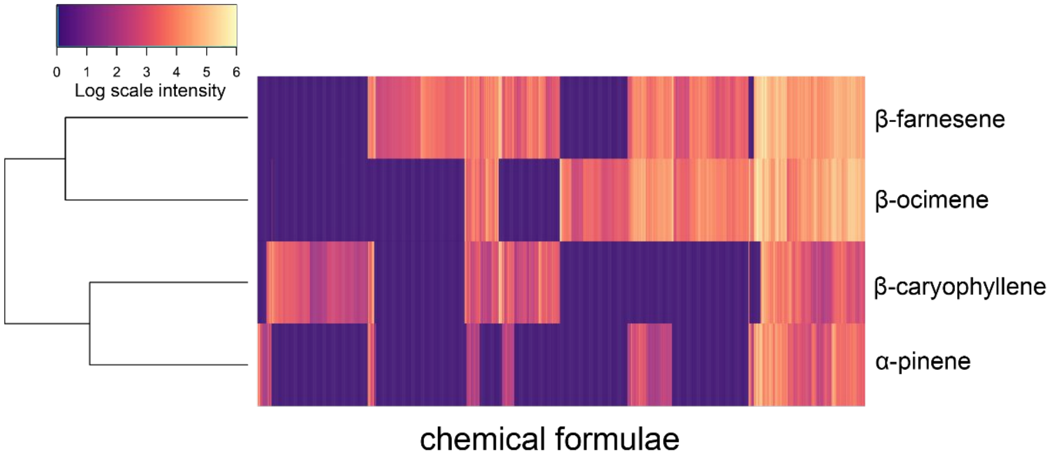

Figure S8 Heatmap comparison of high-resolution mass spectra from four SOA samples. Each vertical strip corresponds to aligned formula, while the rows represent individual samples. Each cell corresponds to the signal intensity of a particular formula in a given sample, with color ranging from low (dark purple) to high (yellow). The heatmap highlights groups of formulas that co-occur at high abundance in specific

samples and help visualize the comparison of the composition of the different SOA systems. Samples with similar intensities for the same formulas were clustered. Distances were calculated from the normalized peak-intensity vectors using Euclidean distance, and clusters were formed using complete-linkage hierarchical clustering.

Table S4. Fitted parameters α-values using the 7-bin volatility basis set (VBS) derived from evaporation kinetics dry experiments for four terpene compounds: α-pinene, β-ocimene, β-caryophyllene, and β-farnesene. The α-values represent the mass fraction yields of the oxidation products in each of the c* bins.

**C*(μg/m3)**	**0.0001**	**0.001**	**0.01**	**0.1**	**1**	**10**	**100**
**α-pinene**	2.52E-01	1.57E-04	1.64E-01	3.60E-01	2.21E-01	1.42E-03	1.58E-04
**β-ocimene**	2.12E-01	2.65E-01	1.14E-01	2.53E-01	1.00E-01	3.36E-02	2.23E-02
**β-caryophyllene**	5.16E-01	1.95E-03	9.71E-02	2.01E-01	1.45E-01	1.58E-02	2.28E-02
**β-farnesene**	6.36E-01	7.81E-03	1.13E-01	1.58E-01	5.51E-02	2.29E-02	7.12E-03

Table S5. Fitted parameters α-values using the 7-bin volatility basis set (VBS) derived from evaporation kinetics wet experiments for four terpene compounds: α-pinene, β-ocimene, β-caryophyllene, and β-farnesene. The α-values represent the mass fraction yields of the oxidation products in each of the c* bins.

**C*(μg/m3)**	**0.0001**	**0.001**	**0.01**	**0.1**	**1**	**10**	**100**
**α-pinene**	2.20E-01	4.44E-03	1.05E-01	4.25E-01	2.37E-01	8.05E-03	7.51E-04
**β-ocimene**	3.54E-01	1.45E-02	1.83E-01	3.29E-01	1.02E-01	8.03E-03	9.00E-03
**β-caryophyllene**	2.54E-01	2.43E-01	7.09E-02	2.84E-01	3.98E-02	1.04E-01	4.48E-03
**β-farnesene**	5.23E-01	7.52E-02	1.49E-01	1.54E-01	7.06E-02	1.77E-02	1.02E-02

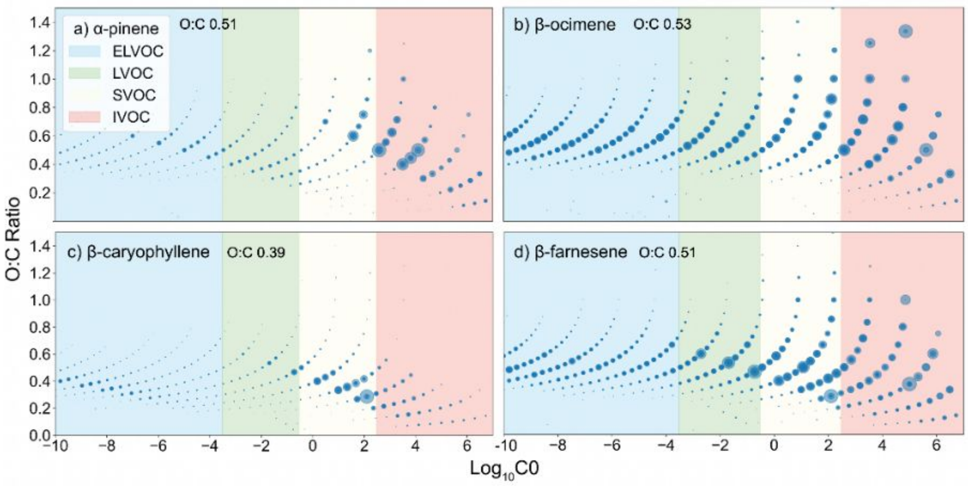

Figure S9. The correlation between the oxygen-to-carbon (O:C) ratio and the volatility, indicated by the log10C0. These estimations are based on chemical formulae identified through nano-DESI-HRMS analysis for SOA originating from OH oxidation of (a) α-pinene, (b) β-ocimene, (c) β-caryophyllene, and (d) β-farnesene. The bubble size within the graph reflects the logarithmic scale of the normalized intensity, offering a visual representation of compound abundance.

References

(1) Roach, P. J.; Laskin, J.; Laskin, A. Molecular Characterization of Organic Aerosols Using Nanospray-Desorption/Electrospray Ionization-Mass Spectrometry. *Anal. Chem.* **2010**, *82* (19), 7979–7986. https://doi.org/10.1021/ac101449p.

(2) Schum, S. K.; Brown, L. E.; Mazzoleni, L. R. MFAssignR: Molecular Formula Assignment Software for Ultrahigh Resolution Mass Spectrometry Analysis of Environmental Complex Mixtures. *Environ. Res.* **2020**, *191*, 110114. https://doi.org/10.1016/J.ENVRES.2020.110114.

(3) Vandergrift, G. W.; Dexheimer, D. N.; Zhang, D.; Cheng, Z.; Lata, N. N.; Rogers, M. M.; Shrivastava, M.; Zhang, J.; Gaudet, B. J.; Mei, F.; China, S. Tethered Balloon System and High-Resolution Mass Spectrometry Reveal Increased Organonitrates Aloft Compared to the Ground Level. *Environ. Sci. Technol.* **2024**, *58* (23), 10060–10071. https://doi.org/10.1021/acs.est.4c02090.

(4) Vandergrift, G. W.; Shawon, A. S. M.; Dexheimer, D. N.; Zawadowicz, M. A.; Mei, F.; China, S. Molecular Characterization of Organosulfate-Dominated Aerosols over Agricultural Fields from the Southern Great Plains by High-Resolution Mass Spectrometry. *ACS Earth Space Chem.* **2022**, *6* (7), 1733–1741. https://doi.org/10.1021/acsearthspacechem.2c00043.

(5) Shannon, C. E. A Mathematical Theory of Communication. *Bell Syst. Tech. J.* **1948**, *27* (3), 379–423. https://doi.org/10.1002/j.1538-7305.1948.tb01338.x.

(6) Wilson, J.; Imre, D.; Beránek, J.; Shrivastava, M.; Zelenyuk, A. Evaporation Kinetics of Laboratory-Generated Secondary Organic Aerosols at Elevated Relative

Humidity. *Environ. Sci. Technol.* **2015**, *49* (1), 243–249. https://doi.org/10.1021/es505331d.
(7) Vaden, T. D.; Imre, D.; Beránek, J.; Shrivastava, M.; Zelenyuk, A. Evaporation Kinetics and Phase of Laboratory and Ambient Secondary Organic Aerosol. *Proc. Natl. Acad. Sci.* **2011**, *108* (6), 2190–2195. https://doi.org/10.1073/pnas.1013391108.
(8) Shrivastava, M.; Zelenyuk, A.; Imre, D.; Easter, R.; Beranek, J.; Zaveri, R. A.; Fast, J. Implications of Low Volatility SOA and Gas-Phase Fragmentation Reactions on SOA Loadings and Their Spatial and Temporal Evolution in the Atmosphere. *J. Geophys. Res. Atmospheres* **2013**, *118* (8), 3328–3342. https://doi.org/10.1002/jgrd.50160.
(9) DeRieux, W. S. W.; Li, Y.; Lin, P.; Laskin, J.; Laskin, A.; Bertram, A. K.; Nizkorodov, S. A.; Shiraiwa, M. Predicting the Glass Transition Temperature and Viscosity of Secondary Organic Material Using Molecular Composition. *Atmospheric Chem. Phys.* **2018**, *18* (9), 6331–6351. https://doi.org/10.5194/ACP-18-6331-2018.
(10) Gordon, M.; Taylor, J. S. Ideal Copolymers and the Second-Order Transitions of Synthetic Rubbers. i. Non-Crystalline Copolymers. *J. Appl. Chem.* **2007**, *2* (9), 493–500. https://doi.org/10.1002/JCTB.5010020901.
(11) Dette, H. P.; Qi, M.; Schröder, D. C.; Godt, A.; Koop, T. Glass-Forming Properties of 3-Methylbutane-1,2,3-Tricarboxylic Acid and Its Mixtures with Water and Pinonic Acid. *J. Phys. Chem. A* **2014**, *118* (34), 7024–7033. https://doi.org/10.1021/JP505910W.
(12) Koop, T.; Bookhold, J.; Shiraiwa, M.; Pöschl, U. Glass Transition and Phase State of Organic Compounds: Dependency on Molecular Properties and Implications for Secondary Organic Aerosols in the Atmosphere. *Phys. Chem. Chem. Phys.* **2011**, *13* (43), 19238–19255. https://doi.org/10.1039/C1CP22617G.
(13) Zobrist, B.; Marcolli, C.; Pedernera, D. A.; Koop, T. Do Atmospheric Aerosols Form Glasses? *Atmospheric Chem. Phys.* **2008**, *8* (17), 5221–5244. https://doi.org/10.5194/ACP-8-5221-2008.
(14) Asa-Awuku, A.; Engelhart, G. J.; Lee, B. H.; Pandis, S. N.; Nenes, A. Relating CCN Activity, Volatility, and Droplet Growth Kinetics of β-Caryophyllene Secondary Organic Aerosol. *Atmospheric Chem. Phys.* **2009**, *9* (3), 795–812. https://doi.org/10.5194/acp-9-795-2009.
(15) Frosch, M.; Bilde, M.; Nenes, A.; Praplan, A. P.; Jurányi, Z.; Dommen, J.; Gysel, M.; Weingartner, E.; Baltensperger, U. CCN Activity and Volatility of β-Caryophyllene Secondary Organic Aerosol. *Atmospheric Chem. Phys.* **2013**, *13* (4), 2283–2297. https://doi.org/10.5194/acp-13-2283-2013.
(16) Smith, N. R.; Crescenzo, G. V.; Huang, Y.; Hettiyadura, A. P. S.; Siemens, K.; Li, Y.; Faiola, C. L.; Laskin, A.; Shiraiwa, M.; Bertram, A. K.; Nizkorodov, S. A. Viscosity and Liquid–Liquid Phase Separation in Healthy and Stressed Plant SOA. *Environ. Sci. Atmospheres* **2021**, *1* (3), 140–153. https://doi.org/10.1039/D0EA00020E.
(17) Wang, J.; Shilling, J. E.; Liu, J.; Zelenyuk, A.; Bell, D. M.; Petters, M. D.; Thalman, R.; Mei, F.; Zaveri, R. A.; Zheng, G. Cloud Droplet Activation of Secondary Organic Aerosol Is Mainly Controlled by Molecular Weight, Not Water Solubility. *Atmospheric Chem. Phys.* **2019**, *19* (2), 941–954. https://doi.org/10.5194/acp-19-941-2019.

(18) Varutbangkul, V.; Brechtel, F. J.; Bahreini, R.; Ng, N. L.; Keywood, M. D.; Kroll, J. H.; Flagan, R. C.; Seinfeld, J. H.; Lee, A.; Goldstein, A. H. Hygroscopicity of Secondary Organic Aerosols Formed by Oxidation of Cycloalkenes, Monoterpenes, Sesquiterpenes, and Related Compounds. *Atmospheric Chem. Phys.* **2006**, *6* (9), 2367–2388. https://doi.org/10.5194/acp-6-2367-2006.

(19) Zhao, D. F.; Buchholz, A.; Tillmann, R.; Kleist, E.; Wu, C.; Rubach, F.; Kiendler-Scharr, A.; Rudich, Y.; Wildt, J.; Mentel, T. F. Environmental Conditions Regulate the Impact of Plants on Cloud Formation. *Nat. Commun.* **2017**, *8* (1), 14067. https://doi.org/10.1038/ncomms14067.

(20) Li, Y.; Pöschl, U.; Shiraiwa, M. Molecular Corridors and Parameterizations of Volatility in the Chemical Evolution of Organic Aerosols. *Atmospheric Chem. Phys.* **2016**, *16* (5), 3327–3344. https://doi.org/10.5194/ACP-16-3327-2016.
